# How I Investigate Bleeding Disorders of Unknown Cause: Current Diagnostic Pathways and Gaps in Laboratory Investigation

**DOI:** 10.1111/ijlh.70191

**Published:** 2026-07-08

**Authors:** Dino Mehic, Faris Karaselimovic, Tim Dreier, Johanna Gebhart

**Affiliations:** ^1^ Division of Hematology and Hemostaseology, Department of Medicine I Medical University of Vienna Vienna Austria

**Keywords:** blood coagulation disorders, blood platelet disorders, hemorrhagic disorders, inherited, platelet function tests, von Willebrand diseases

## Abstract

Bleeding disorder of unknown cause (BDUC) constitutes the largest group of patients presenting with a mild‐to‐moderate bleeding tendency in tertiary care settings. Their clinical bleeding phenotype is characterized by mucocutaneous bleeding, as well as bleeding following surgical challenges or childbirth, and is associated with impaired health‐related quality of life. Iron deficiency, with or without anemia, is common, particularly among women, who comprise up to 80% of BDUC cohorts and frequently report heavy menstrual bleeding. Diagnosing BDUC requires a rigorous exclusion of established hemostatic and non‐hemostatic causes of bleeding. Common inherited bleeding disorders, including coagulation factor deficiencies (CFD), von Willebrand disease (VWD), and platelet function disorders (PFD), must be systematically excluded. CFD evaluation should extend beyond routine assays (prothrombin time, activated thromboplastin time, thrombin time), as clinically relevant mild reductions in factors VIII, IX, and XI may occur despite normal screening tests; and factor XIII deficiency is not detected by global assays. VWD assessment mandates measurement of VWF antigen and activity, with additional studies to define subtype when indicated. For PFD, light transmission aggregometry remains the reference gold standard. Substantial diagnostic overlap exists among these entities and BDUC, and repeated testing is often required. Investigations for rare causes such as hyperfibrinolysis or excess natural anticoagulants are typically limited to patients with distinctive phenotypes or strong family histories. Although the pathogenesis of BDUC remains incompletely understood, continued investigation into platelet biology, global hemostasis, and vascular contributions holds promise for uncovering therapeutic targets, ultimately improving management for this prevalent yet understudied condition.

## 1 Introduction

Patients with a clinically relevant mild‐to‐moderate bleeding tendency in whom no specific hemostatic abnormality can be identified are classified as having a bleeding disorder of unknown cause (BDUC) [1, 2, 3]. The diagnosis of BDUC accounts for up to two‐thirds of adults with a mild‐to‐moderate bleeding tendency who present to tertiary care centers. Common symptoms include easy bruising, heavy menstrual bleeding, and prolonged bleeding following trauma or surgical procedures [3]. Although bleeding is often perceived as mild‐to‐moderate, it can be severe and occasionally life‐threatening, particularly in the context of surgery or childbirth [3]. The majority of affected patients are women, who frequently exhibit iron deficiency, and overall, patients with BDUC experience impaired health‐related quality of life (Figure 1) [5, 6].

**FIGURE 1:**
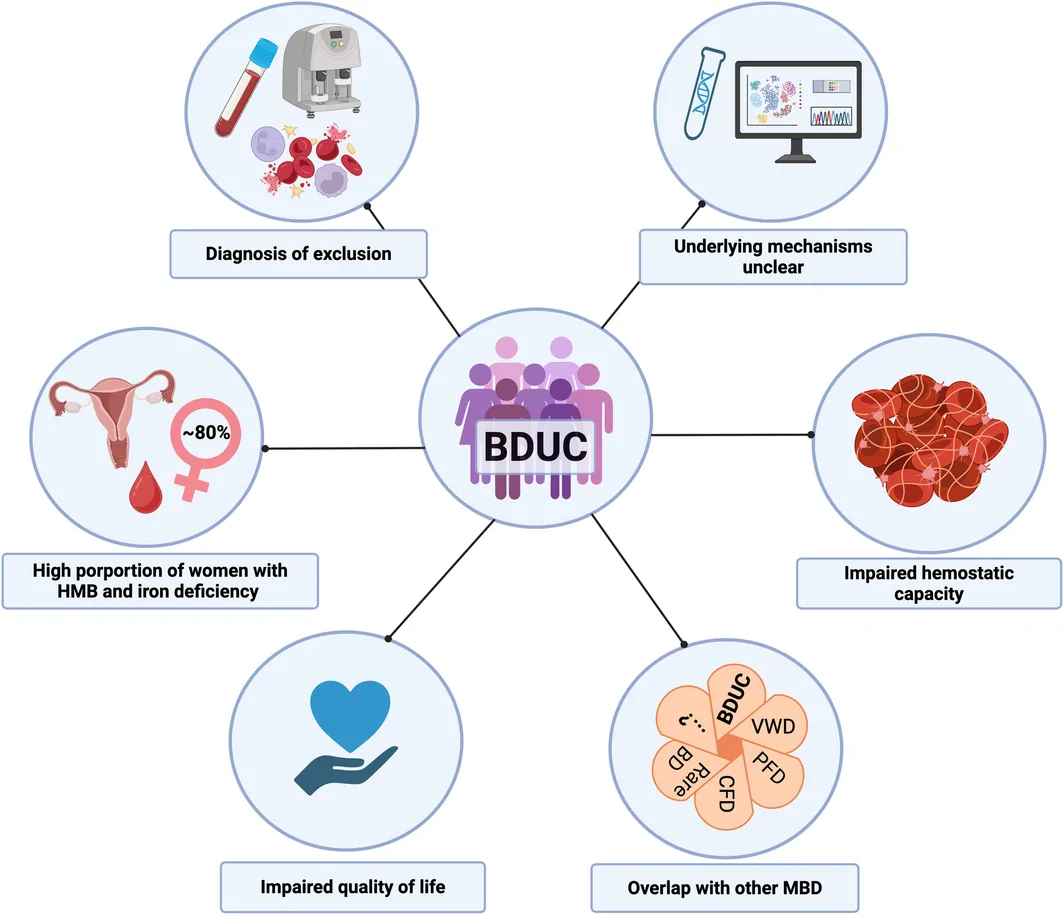
Clinical presentation and current diagnostic challenges of patients with BDUC MBD, mild‐to‐moderate bleeding disorders. BD, bleeding disorder; BDUC, bleeding disorder of unknown cause; CFD, coagulation factor deficiency; HMB, heavy menstrual bleeding; PFD, platelet function defect; VWD, von Willebrand disease. Created in BioRender. Mehic [4] https://BioRender.com/kuux4nm.

After ruling out acquired or inherited vascular causes of bleeding such as connective tissue disorders, among other differential diagnoses, a comprehensive laboratory evaluation of plasmatic coagulation and platelet function is required to exclude known coagulation disorders in a patient [1, 7]. The Scientific and Standardization Committee (SSC) of the International Society on Thrombosis and Haemostasis (ISTH) has proposed a standardized diagnostic framework that includes a broad panel of coagulation and platelet assays [8]. Depending on local expertise and resources, additional investigations assessing natural anticoagulant pathways, fibrinolytic activity, or endothelial contributions to hemostasis may be considered [2, 8, 9]. While global hemostatic assays have provided important insights into disease mechanisms, they are currently not recommended for routine diagnostic evaluation [10].

To study the natural history of BDUC more comprehensively, the Vienna Bleeding Biobank was established in 2009. In this observational prospective cohort study, we enroll adult patients with a clinically relevant bleeding tendency who have previously not been investigated for a bleeding disorder [3, 11].

In this review, we summarize the current laboratory diagnostic approach and its challenges in the evaluation of BDUC, a diagnosis of exclusion, and discuss specialized and emerging assays that may help refine phenotyping and advance our understanding of bleeding mechanisms in this heterogeneous patient population.

## 2 Diagnostic Work‐Up of BDUC

Distinguishing between trivial and clinically relevant bleeding can be challenging and requires substantial clinical expertise to interpret a patient's bleeding tendency [2, 12]. On the other hand, standardized bleeding assessment tools (BATs), such as the ISTH‐BAT, are recommended in this process; however, a pathological bleeding score is not necessarily required, as bleeding scores are influenced by age, sex‐specific symptoms, and the cumulative number of hemostatic challenges encountered over a lifetime [13]. Importantly, clinicians should also consider hereditary causes unrelated to bleeding disorders, such as connective tissue disorders (e.g., patients with signs of hypermobility), as well as acquired bleeding tendencies due to medications or dietary supplements and medical conditions such as renal or thyroid disease (Figure 2) [7, 8].

**FIGURE 2:**
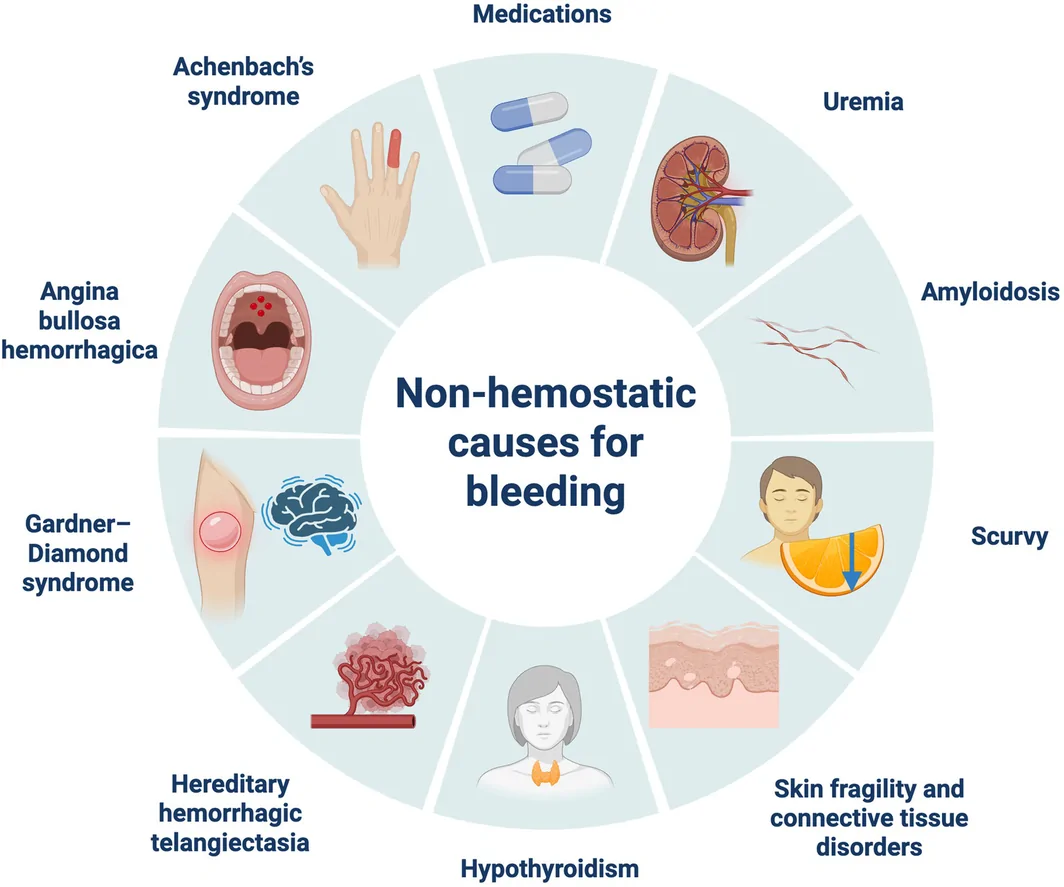
Non‐hemostatic causes for bleeding in the diagnostic work‐up of patients with bleeding disorder of unknown cause. Created in BioRender. Mehic [14] https://BioRender.com/q97aan5.

Next, the diagnosis of BDUC requires a structured and comprehensive laboratory evaluation. The suggested panels to study plasmatic coagulation and platelet function testing according to the ISTH guidance are summarized in Table 1 [8]. All patients should undergo a complete blood count, activated partial thromboplastin time (aPTT), prothrombin time (PT), thrombin time (TT), and fibrinogen measurement. Measurement of coagulation factors VIII, IX, and XI is recommended even in the presence of a normal aPTT, as mild deficiencies may not be detected. Abnormalities in global coagulation assays could prompt targeted measurement of individual coagulation factors (including factors II, V, VII, X), in the case of high clinical suspicion. This approach also extends to factor XIII deficiency, which does not affect PT or aPTT and is exceedingly rare in this population [3, 15].

**TABLE 1:** Laboratory evaluation of bleeding disorder of unknown cause.

Complete blood count and coagulation assays
PT, aPTT
Fibrinogen Clauss
Factor VIII, IX, XI activity^a^
Von Willebrand factor testing: antigen and activity^b^
Advanced VWF investigations to define VWD subtype: VWF multimer analysis, VWF collagen‐binding assay, ristocetin‐induced platelet aggregation, FVIII‐VWF binding assay, and genetic testing where appropriate
Platelet function testing
Light transmission aggregometry (gold standard)
Platelet secretion assays^c^

Abbreviations: aPTT, activated partial thromboplastin time; CBC, complete blood count; PT, prothrombin time.

^a^ Factor VIII, IX, XI should be assessed regardless of aPTT time. Suspected patients with BDUC may progress to other specific investigations of rare coagulation factor deficiencies (including factors II, V, VII, X, and XIII) if available.

^b^ Platelet‐dependent VWF activity assays (e.g., VWF:GPIbM, VWF:GPIbR) are recommended over VWF:RCo where available.

^c^ Platelet secretion assays such as glycoprotein expression, evaluations for dense granule deficiency and/or impaired secretion release (e.g., lumi‐aggregometry measuring adenosine triphosphate release from dense granules, whole‐mount transmission platelet electron microscopy to quantify platelet dense granules or an assessment of mepacrine release from dense granules) should ideally be performed according to the ISTH guidelines to exclude a platelet‐dense granule deficiency before BDUC is diagnosed. If this is not possible, BDUC should be the diagnosis until proven otherwise, as shown by the dotted green arrow (adapted from the SSC communication).

For the evaluation of von Willebrand disease (VWD), initial assessment should include measurement of von Willebrand factor antigen (VWF:Ag) and platelet‐dependent VWF activity (VWF:Act) assays [8]. For VWF:Act testing, it is recommended to use platelet‐dependent VWF:Act (e.g., VWF:GPIbM, VWF:GPIbR) over the VWF ristocetin cofactor assay (VWF:RCo) [16]. The VWF collagen‐binding assay (VWF:CB) is currently recommended primarily for the classification of type 2 VWD [16]. Its potential utility as a screening tool remains uncertain and warrants further investigation. In addition, further specialized tests can be performed to distinguish between VWD subtype [16], as described below.

In the assessment of platelet function defects (PFD), light transmission aggregometry (LTA) remains the recommended gold standard. In patients with persistent clinical suspicion despite normal aggregation studies, platelet secretion assays such as glycoprotein expression, evaluations for dense granule deficiency and/or impaired secretion release (e.g., lumi‐aggregometry measuring adenosine triphosphate release from dense granules, whole‐mount transmission platelet electron microscopy to quantify platelet dense granules or an assessment of mepacrine release from dense granules) should ideally be performed according to the ISTH guideline [8].

Patients with BDUC may proceed to additional specialized investigations when available. These may include fibrinolysis assays (plasmin generation, euglobulin clot lysis time, plasminogen activator inhibitor‐1, tissue plasminogen activator, and α2‐antiplasmin activity), evaluation for rare clotting factor deficiencies (factors II, V, VII, X, XIII), and highly specialized assays for thrombomodulin, bleeding‐associated FV variants (East Texas and Amsterdam), tissue factor deficiency, and rare platelet disorders such as Scott syndrome and Quebec platelet disorder, as well as targeted genomic testing. Overall, any abnormal test results should be repeated to confirm the diagnosis of specific mild‐to‐moderate bleeding disorder.

In patients with suspected acquired bleeding disorders, particularly in the absence of a lifelong bleeding history, evaluation should extend beyond hemostatic testing to systematically exclude underlying medical conditions. Frequently performed non‐hemostatic laboratory investigations include assessment of liver, renal, and thyroid function. In addition, serum protein electrophoresis can be performed to screen for paraproteinemia. Measurement of C‐reactive protein is recommended as a marker of systemic inflammation and to account for potential acute‐phase–related elevations in factor VIII and von Willebrand factor levels. Finally, serum ferritin should be assessed to evaluate iron stores, which may be depleted as a consequence of chronic or excessive bleeding.

## 3 Challenges and Novel Aspects in the Diagnostic Work‐Up for BDUC

When evaluating patients with a mild‐to‐moderate bleeding tendency, several challenges remain regarding the indication for specialized assays and the definition of appropriate diagnostic cutoffs [8]. As mentioned above, the most common bleeding disorders including CFD, VWD, and PFD must be excluded when diagnosing a patient with BDUC. The following sections discuss diagnostic considerations for each underlying disease in more detail:

### 3.1 Coagulation Factor Deficiencies

Deficiencies of coagulation factors are rare disorders and, when severe, such as in severe hemophilia A or B, typically diagnosed during early childhood [17]. According to ISTH SSC recommendations, factor VIII and factor IX levels below 40% are used as diagnostic cutoffs for hemophilia A or B [18]. However, observational studies by Rejtö et al. and Gebhart et al. suggest that a higher threshold of 50% may be clinically relevant [3, 17]. Importantly, the relationship between factor level and bleeding phenotype is not uniform across all coagulation factors. For factors with a well‐established and strong correlation between activity levels and bleeding severity (e.g., FVIII, FIX), diagnostic thresholds are clinically meaningful and generally predictive of phenotype. In contrast, for other coagulation factors with weaker or more variable genotype–phenotype correlations, the clinical significance of mildly reduced levels is far less clear. In these settings, mild laboratory abnormalities may not fully account for the observed bleeding tendency, and phenotypic overlap with BDUC is likely [19, 20]. This includes assessment of factors II, V, VII, and X [8]. Defining clinically meaningful cutoffs for these very rare coagulation factor deficiencies is challenging, as bleeding severity often correlates poorly with measured factor activity, as previously summarized by Palla et al. [20]. Hypofibrinogenemia can be defined as a fibrinogen concentration ≤ 100 mg/dL. Deficiencies of factors II and V can be defined by activity levels ≤ 10%, and factor VII deficiency by levels ≤ 20%. Factor XI deficiency can be defined using a higher threshold (≤ 60%), reflecting clinical data demonstrating bleeding manifestations even in patients with partial FXI deficiency at or above this level [21]. Factor XIII activity and antigen testing should also be included if available, as factor XIII deficiency represents an important diagnostic blind spot that is not detected by routine global coagulation assays and effective treatment is available. At the same time, data from our group and others demonstrate the limited diagnostic yield of systematic factor XIII testing due to the extreme rarity of the disorder [3, 15].

### 3.2 Von Willebrand Disease

VWD is defined at VWF:Ag and/or VWF:Act levels below 50 IU/dL in the presence of a clinically significant bleeding tendency, according to current guidelines [16]. Individuals with VWF levels below 30 IU/dL can be diagnosed with VWD irrespective of bleeding symptoms. Diagnosis remains challenging because VWF levels correlate only modestly with bleeding severity, particularly in individuals with VWF levels between 30 and 50 IU/dL [16]. Details on laboratory VWD diagnostics have been recently updated [16]. In short, VWD can be broadly classified into quantitative and qualitative defects. Type 1 VWD represents a partial quantitative deficiency, typically with levels below 30 IU/dL or between 30 and 50 IU/dL in combination with a positive history of bleeding, whereas type 3 VWD is characterized by a near complete absence of VWF [16, 22]. Type 2 VWD encompasses qualitative defects and is defined by a reduced VWF:Act to VWF:Ag ratio, generally below 0.7. Type 2 VWD is further subdivided into types 2A, 2B, and 2 M, while type 2 N is characterized by impaired binding of VWF to factor VIII. Accurate subtype classification is clinically important, as treatment strategies differ. For example, desmopressin is contraindicated in type 2B VWD because it may exacerbate thrombocytopenia [23].

Laboratory assessment of VWF is complicated by substantial intra‐ and inter‐individual variability and by multiple biological and preanalytical factors [16, 22]. Estrogen exposure, including during pregnancy or hormone replacement therapy, increases VWF levels, and diagnostic testing should therefore ideally be performed outside of high estrogen states. Cycle‐dependent hormonal fluctuations are generally modest but may be relevant in individuals with values near diagnostic thresholds. VWF increases during acute phase reactions and may be elevated during inflammation, infection, or trauma. In such settings, C‐reactive protein should be assessed and repeat testing after resolution of the acute condition is recommended. Diagnosis is particularly challenging in patients with autoimmune or malignant disease, in whom sustained VWF elevation may obscure underlying defects [22]. VWF levels increase with age in both healthy individuals and patients with type 1 VWD, and higher levels are associated with several comorbid conditions, including atherosclerosis, malignancy, and liver disease [22]. Importantly, increased VWF levels with aging do not necessarily translate into improvement of bleeding symptoms. Atiq et al. recently demonstrated that, in some patients, low VWF levels in the range of 30–50 IU/dL reflect an age‐dependent increase in VWF levels in individuals with type 1 VWD (VWF levels < 30 IU/dL). Moreover, it is suspected that a subset of patients classified as having BDUC may similarly represent patients with VWD who present later in life for clinical workup, at a time point when age‐dependent increases in VWF levels partially mask the underlying disorder [24].

Blood group O is associated with lower VWF levels [22]. Beyond this effect, blood group O has been shown to influence bleeding severity independently of VWF levels and to be associated with increased perioperative and postpartum bleeding risk in patients with mild‐to‐moderate bleeding tendencies [25]. While ABO blood group should be documented during bleeding assessment, it no longer plays a role in the formal laboratory diagnosis of VWD [16].

In the Vienna Bleeding Biobank, repeated testing showed in 13% of 277 patients fluctuating VWF levels across the 50 IU/dL diagnostic threshold, with nearly one quarter of VWD diagnoses established only at follow‐up [26]. Similar observations in pediatric and adolescent bleeding cohorts underscore the limitations of single time‐point testing [27]. Based on our prospective data on repeated testing, an 80 IU/dL cutoff yielded a negative predictive value of 98.1% and may allow repeat VWF testing to be restricted to patients below this threshold, reducing diagnostic overlap between low VWF and BDUC.

### 3.3 Platelet Function Defects

The diagnosis of PFD requires specialized expertise and is therefore largely restricted to tertiary care centers with experience in platelet function testing [28]. Severe PFDs, or those associated with syndromic features such as facial dysmorphia or skeletal abnormalities, are typically recognized in early childhood [29, 30]. In contrast, many patients present in adulthood with bleeding symptoms attributable to mild PFDs. In this setting, laboratory abnormalities are often subtle, phenotypes heterogeneous, and there is substantial overlap with BDUC [1].

Pre‐analytical variables including patient‐related factors (e.g., smoking, medications such as selective serotonin reuptake inhibitors, age, comorbidities), timely sample processing, and hematocrit, and strict protocol standardization are critical determinants of platelet function testing and must be carefully controlled to ensure reliable and interpretable results [29]. In general, repeat platelet studies should be separated by at least 1 month to allow disappearance of acquired interfering factors [28].

To diagnose a PFD, a stepwise laboratory testing approach is recommended [28]. As mentioned, LTA remains the reference standard, although its interpretation requires careful consideration of pre‐analytical variables, assay limitations, and clinical context. Recommended agonists and concentrations can be found in the current ISTH guidance [28]. Of note, lower agonist concentrations meeting these benchmarks are increasingly discussed as potentially more sensitive [31]. In mild PFD, subcategorization into distinct pathophysiologic groups based solely on laboratory abnormalities is often challenging, as phenotypes are subtle and overlapping.

If first‐step testing with LTA is normal, second‐step investigations are advised and include extended LTA, flow cytometry, assessment of platelet granule content, serum thromboxane B_2_, clot retraction, and transmission electron microscopy [28]. Indeed, platelet dense granule deficiency is often underdiagnosed due to the lack of widely available and standardized assays. In a recent French study of 119 patients with BDUC, 66 exhibited at least one abnormal dense granule assay, including whole‐mount transmission electron microscopy, mepacrine uptake/release, or CD63 expression [32]. When first‐ and second‐step testing fail to establish a diagnosis but clinical suspicion remains high, molecular genetic testing may be warranted [28].

The platelet function analyzer (PFA‐100/‐200) is not recommended for the diagnosis of PFDs, as its performance is highly dependent on VWF levels as well as hematocrit and cannot reliably distinguish PFD from BDUC, as previously shown by our group [28, 33]. Nevertheless, platelet dysfunction beyond abnormalities detected by light transmission aggregometry is suspected in some BDUC patients. For example, impaired procoagulant platelet formation has been reported in BDUC [34]. Supporting these observations, Heubel‐Moenen et al. investigated 14 BDUC patients with prolonged PFA closure times using microfluidic flow chambers and demonstrated reduced platelet adhesion and impaired thrombus formation under shear compared with healthy controls [35].

Despite advances in molecular diagnostics, the underlying cause of platelet dysfunction remains unidentified in approximately one‐third of patients with suspected PFD [29]. Data from the Vienna Bleeding Biobank suggest that impaired platelet maturation, reflected by reduced glycoprotein expression on immature platelets, may contribute to bleeding risk [36]. More recently, altered platelet lipid signatures in female patients with PFD were associated with impaired aggregation and predicted responses to ADP and TRAP‐6 stimulation, highlighting platelet lipidomics as a potential source of future biomarkers and therapeutic targets [37].

### 3.4 Other Rare Bleeding Disorders

In patients with a strong bleeding history but inconclusive standard testing, rare hemostatic disorders that phenotypically overlap with BDUC may need to be considered [1].

Over the past two decades, particular attention has been directed toward disorders driven by enhanced natural anticoagulant activity, which can result in clinically relevant bleeding despite largely normal routine coagulation assays [38]. Several gain of function variants in exon 13 of the factor V gene (F5) have been described, including those underlying the East Texas bleeding disorder as well as the FV Amsterdam and FV Atlanta bleeding disorders [39, 40, 41]. These variants promote better interaction between factor V and tissue factor pathway inhibitor alpha (TFPIα), leading to an increased half‐life of TFPIα and suppression of thrombin generation [42]. Clinically, affected individuals present with mucocutaneous and procedure‐related bleeding that may resemble BDUC. In contrast to most patients with BDUC, laboratory evaluation often reveals prolonged PT and aPTT. Previous data from our and other groups suggest that increased TFPI levels may represent a modifying factor for bleeding in subsets of patients with BDUC and in individuals with factor XI deficiency [43, 44, 45].

A distinct mechanism of bleeding has been identified in single cases and families with thrombomodulin associated coagulopathy [46, 47, 48]. Pathogenic variants in the THBD gene can increase endothelial shedding of thrombomodulin, resulting in elevated circulating soluble thrombomodulin and enhanced activation of the protein C pathway. This condition has been associated with bleeding complications, particularly following surgical interventions or trauma, and in rare cases with spontaneous intra‐abdominal bleeding. Although routine coagulation tests and platelet function studies are typically unremarkable, evaluation of soluble thrombomodulin with conventional ELISA may provide diagnostic insight. In our cohort within the Vienna bleeding biobank, however, soluble thrombomodulin concentrations did not differ between patients with BDUC or other mild bleeding disorders and healthy controls, and no association with bleeding severity or global coagulation assays was observed. Moreover, no pathogenic THBD variants were detected [49].

Another exceptionally rare cause of bleeding is the α1 antitrypsin Pittsburgh variant, which arises from a Met358Arg substitution. This single amino acid change transforms α1 antitrypsin into a potent inhibitor of thrombin and contact pathway proteases and is associated with a severe systemic bleeding phenotype [50].

More recently, impaired tissue factor expression has been implicated as a cause of severe unexplained bleeding. A heterozygous frameshift variant in the tissue factor gene (F3) was shown to result in premature truncation of the protein, producing a shortened tissue factor isoform referred to as TF short [51].

Finally, dysregulation of fibrinolysis should be considered in patients with otherwise unexplained bleeding, particularly when bleeding is delayed or disproportionate to the inciting trigger, as previously summarized by our group and Saes et al. [9, 52]. Monogenic hyperfibrinolytic disorders are very rare and frequently associated with consanguinity, but several well‐characterized entities exist [9, 52]. These include deficiencies of α2 antiplasmin and plasminogen activator inhibitor 1, excess tissue type plasminogen activator, and Quebec platelet disorder. The latter is caused by a tandem duplication of the PLAU gene that includes a downstream enhancer that is repositioned and rewires PLAU, causing markedly increased PLAU expression selectively by megakaryocytes and increased storage of urokinase type plasminogen activator within platelet alpha granules, which clinically manifests as delayed bleeding [53].

## 4 Genetic Testing

Genetic testing has been proposed as a diagnostic tool in patients with BDUC. However, results from the Thrombogenomics project, which analyzed more than 600 patients with BDUC, including participants from the Vienna Bleeding Biobank, demonstrated a low diagnostic yield: [54] whole exome sequencing of over 96 genes involved in plasmatic coagulation and platelet function identified pathogenic variants in only approximately 3% of cases. These data suggest that BDUC more commonly reflects the combined effects of multiple modest genetic factors rather than a single monogenic defect [54].

Nonetheless, rare monogenic causes cannot be excluded. Emerging evidence indicates that variants in genes not traditionally associated with hemostasis may contribute to bleeding phenotypes in selected patients. For example, mutations in *APOLD1* have been shown to impair endothelial barrier function by disrupting the junction‐cytoskeletal interface, resulting in increased endothelial permeability and spontaneous release of Weibel‐Palade body contents, including VWF and angiopoietin‐2 [55].

## 5 Global Hemostatic Assays

Global assays have been proposed as complementary tools for hemostatic evaluation in patients with BDUC, as previously summarized [10]. Previous studies from our group and others have demonstrated impaired thrombin generation and plasmin generation in BDUC, supporting the concept of a reduced global hemostatic capacity in a subset of patients [56, 57, 58]. However, these plasmatic assays have not been incorporated into routine diagnostic algorithms and currently remain primarily research tools used to characterize global coagulation and fibrinolytic potential [8]. Viscoelastic testing has also been explored in BDUC patients [4, 59]. Data from the Vienna Bleeding Biobank demonstrate that assays such as ROTEM can distinguish patients with BDUC from healthy controls [4]. In addition, Monard et al. showed that an adapted tPA‐ROTEM assay can identify hyperfibrinolytic profiles in a clinically relevant proportion of BDUC patients, highlighting fibrinolysis as a potential contributor to bleeding in this population [59].

## 6 Future Directions

Despite significant advances in the diagnostic work‐up of BDUC patients, several key uncertainties remain:

- A central challenge is the definition of a clinically relevant bleeding phenotype: it is still sometimes unclear which degree and pattern of bleeding should qualify for a diagnosis of BDUC, and whether current assessment tools such as bleeding scores adequately capture disease burden across diverse patient populations [14].
- The role of genetic testing remains to be defined, particularly in the context of expanding gene panels with uncertain diagnostic yield in mild bleeding phenotypes [54, 60].
- Similarly, the potential of global hemostatic assays to improve diagnostic sensitivity and better reflect the in vivo hemostatic balance is promising but not yet established for routine clinical use [10].
- Further controversy exists regarding the sensitivity of current platelet function testing, raising the possibility that mild platelet function defects remain undetected. Furthermore, the role of reduced procoagulant platelet formation or impaired shear‐dependent platelet function is being investigated [34, 35].
- In addition, some patients classified as BDUC may represent evolving von Willebrand disease, with VWF levels above current diagnostic thresholds, or undiagnosed hemophilia carriers with subtle phenotypes [24, 61, 62].

Addressing these gaps will require integrative approaches combining detailed phenotyping with advanced laboratory methods, including multi‐omics and functional assays, to refine disease classification and ultimately enable more precise, mechanism‐based management strategies.

## 7 Conclusions

Patients with BDUC constitute the largest group among individuals presenting with mild‐to‐moderate bleeding tendencies in tertiary care settings. Within the Vienna Bleeding Biobank, and in alignment with SSC ISTH recommendations, BDUC is diagnosed using a structured, pragmatic approach. This process relies on the systematic exclusion of non‐hemostatic causes of bleeding as well as established bleeding disorders, including CFD, VWD, and PFD. Nevertheless, some rare bleeding disorders may remain undetected with standard testing, underscoring the need for targeted investigations in patients with suggestive clinical phenotypes.

Despite the absence of an unifying laboratory diagnosis, patients with BDUC share a clinically meaningful bleeding phenotype, remain at risk for future bleeding, frequently develop iron deficiency with or without anemia, and experience substantial impairment in health‐related quality of life. Accordingly, BDUC should be considered in the differential diagnosis of all patients referred for evaluation of a bleeding tendency, and management should extend beyond diagnostic labeling to include individualized care plans, particularly in anticipation of future hemostatic challenges.

## Author Contributions

Dino Mehic, Faris Karaselimovic, Tim Dreier, and Johanna Gebhart wrote and revised the manuscript.

## Funding

The authors have nothing to report.

## Conflicts of Interest

Dino Mehic received honoraria for advisory board meetings and lectures from CSL Behring and SOBI, a research grant from CSL Behring (Prof. Heimburger award) and travel support by Sobi, Novo Nordisk, Pfizer, and Roche. Faris Karaselimovic has nothing to declare. Tim Dreier received honoraria for advisory board meetings and lectures from CSL Behring and a research grant from CSL Behring (Prof. Heimburger award). Johanna Gebhart received research grants from CSL Behring, Sobi, Takeda, Amgen, and Novartis for the Medical University of Vienna; honoraria for lectures and advisory board meetings from CSL Behring, Sobi, Novartis, and Amgen.

## Data Availability

Data sharing not applicable to this article as no datasets were generated or analysed during the current study.
